# Adult-Onset Still's Disease Following COVID-19 Infection in a Patient Receiving Nirmatrelvir/Ritonavir: A Case Report

**DOI:** 10.7759/cureus.60946

**Published:** 2024-05-23

**Authors:** Georges El Hasbani, Andres I Applewhite, William Scheuing, Lawena Maher

**Affiliations:** 1 Medicine, St. Vincent's Medical Center, Bridgeport, USA; 2 Internal Medicine, Mayo Clinic, Jacksonville, USA; 3 Medicine, Brown University, Providence, USA

**Keywords:** interleukin-1, inflammatory cytokines, treatment, adult-onset still's disease, covid-19

## Abstract

SARS-CoV-2 (COVID-19) has been associated with numerous complications, including autoimmune and autoinflammatory diseases. The surge of cytokines following COVID-19 infection or vaccination has been proposed to contribute to immune dysregulation, which might subsequently give rise to an autoinflammatory syndrome. Adult-onset Still's disease (AOSD) is one of the rare autoinflammatory diseases characterized by a surge of cytokines. Although an association between COVID-19 vaccines and AOSD has been reported, an association with COVID-19 infection or nirmatrelvir/ritonavir remains very rare. In this case, we present a patient who developed AOSD after COVID-19 infection and subsequent treatment with nirmatrelvir/ritonavir. After the initial response to glucocorticoids, canakinumab was initiated, resulting in positive clinical outcomes.

## Introduction

Severe acute respiratory syndrome coronavirus 2 (SARS-CoV-2), an enveloped, positive-sense single-stranded genomic RNA virus [1], has emerged as one of the greatest threats to public health in the 21st century [2]. Due to its significant morbidity and mortality, various antivirals, monoclonal antibodies, and immunomodulatory drugs have been tried as treatment approaches. However, most of these measures have not been effective [3]. A combination of nirmatrelvir and ritonavir has shown promising results in reducing severe COVID-19 mortality [3, 4].

SARS-CoV-2 can affect a variety of immune cells, leading to massive cytokine release [3]. Such immune system alterations can result in immune dysregulation syndromes [5] In rare cases, nirmatrelvir/ritonavir has been associated with the induction or exacerbation of certain autoimmune or autoinflammatory diseases, despite lacking a clearly defined pathophysiology [6]. Adult-onset Still's disease (AOSD) is a systemic inflammatory disease characterized by a surge of cytokines such as interleukin (IL)-1, IL-6, IL-18, and IL-37 [7]. The primary clinical features of AOSD include spiking fever, arthritis, and salmon-colored skin rash [8]. Diagnosis is typically based on clinical evaluation, although certain laboratory markers such as ferritin levels can be suggestive. Although possible, the association between COVID-19 and AOSD is rarely reported. AOSD may occur after COVID-19 infection or COVID-19 vaccination, but there have been no reports following nirmatrelvir/ritonavir treatment.

In this report, we present a case of AOSD that developed after COVID-19 infection and treatment with nirmatrelvir/ritonavir. Our patient was treated with high-dose glucocorticoids and canakinumab, leading to positive clinical outcomes and disease control.

## Case presentation

**Figure 1:**
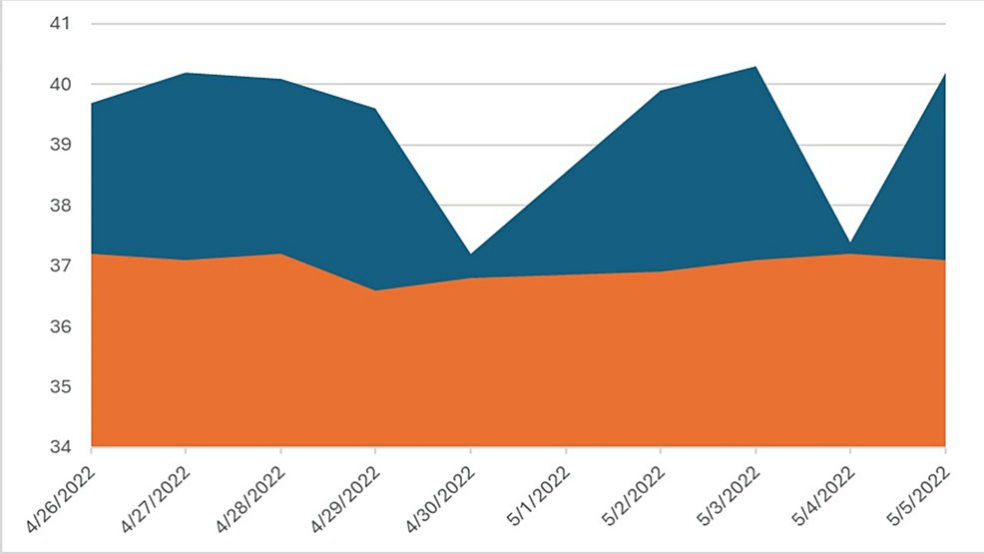
Fever curve

A 55-year-old female with a medical history of non-insulin-dependent diabetes, hypothyroidism, hypertension, hyperlipidemia, and morbid obesity (BMI of 47.13) presented to the emergency department with a two-week history of malaise, odynophagia, fever, and myalgia. Notably, she was hospitalized for COVID-19 pneumonia the month prior to presentation and received treatment with nirmatrelvir/ritonavir. She was experiencing progressively worsening sharp neck pain and sore throat exacerbated by movement. Fevers were quotidian and later progressed to double quotidian (maximum temperature 104 °F) (FIgure 1).

On examination, cervical lymphadenopathy was noted. No generalized rashes were present. Lungs were clear. Cardiovascular examination was negative for murmurs or pericardial knocks. Blood investigations showed leukocytosis with a left shift, elevated erythrocyte sedimentation rate (ESR), C-reactive protein (CRP), and ferritin (Table 1).

**Table 1:** Blood investigations at presentation. ALT - Alanine Aminotransferase; AST - Aspartate Aminotransferase; PT - Prothrombin Time; PTT - Partial Thromboplastin Time; CRP - C-Reactive Protein; ESR - Erythrocyte Sedimentation Rate; LDH - Lactate Dehydrogenase; ANA - Antinuclear Antibody; ANCA - Antineutrophil Cytoplasmic Antibodies; dsDNA - Double-stranded DNA; TSH - Thyroid Stimulating Hormone; T4 - Thyroxine; Ig: immunoglobulin; IL: interleukin; PCR: Polymerase Chain Reaction

Parameter	Results	Normal Range
White Blood Cells	15.1 × 10^9^/L	3.5 – 11.0 x 10^9^/L
Absolute Neutrophil Count	12.6 × 10^9^/L	1.5 – 7.5 x 10^9^/L
Hemoglobin	13.2 g/dL	11.0-15.0 g/dL
Platelets	311 × 10^9^/L	150-400 × 10^9^/L
Creatinine	0.76 mg/dL	0.44-1.03 mg/dL
Alkaline Phosphatase	111 IU/L	34 – 104 IU/L
Total Bilirubin	0.6 mg/dL	0.2 – 1.3 mg/dL
ALT	21 IU/L	6 – 45 IU/L
AST	29 IU/L	10 – 42 IU/L
Fasting Triglycerides	118 mg/dL	40 – 149 mg/dL
PT	15.6 sec	11 – 13.5 sec
PTT	28.0 sec	25 – 35 sec
Procalcitonin	0.38 ng/mL	< 0.1 ng/mL
CRP	320 mg/L	0 – 10.00 mg/L
ESR	> 130 mm/H	0 – 30 mm/H
Ferritin	6,354 ng/mL	10 – 120 ng/mL
D dimer	859 ng/mL	0 – 300 ng/mL
Fibrinogen level	565 mg/dL	40 – 149 mg/dL
LDH	269 IU/L	100 – 220 IU/L
Creatine Kinase	31 IU/L	20 – 165 IU/L
ANA	Positive 1:40	NA
ANCA	Negative	NA
dsDNA	2 IU/mL	0 – 4 IU/mL
Anti-ribonucleoprotein	Negative	NA
TSH	8.116 uIU/mL	0.35 – 5.50 uIU/mL
Free T4	1.02 ng/dL	0.9 – 2.3 ng/dL
Blood Cultures	No Growth after 5 days	NA
Hepatitis A virus IgM Ab	Non-reactive	NA
Hepatitis B virus S Ag	Non-reactive	NA
Hepatitis B virus Core IgM Ab	Non-reactive	NA
Hepatitis C virus Ab	Non-reactive	NA
TB Quantiferon Gold Plus	Indeterminate	NA
Parvovirus IgM & IgG	Negative	NA
Cytomegalovirus IgM & IgG	Negative	NA
Virus Respiratory Panel	Negative	NA
Lyme Antibodies	Negative	NA
Babesia PCR	Negative	NA
Anaplasma PCR	Negative	NA
Actin Ab IgG	Negative	NA
Anti-mitochondrial Ab	Negative	NA
Rheumatoid Factor	8 IU/mL	< 15 IU/mL
C4 Complement	28 mg/dL	15 – 57 mg/dL
C3 Compliment	191 mg/dL	83 – 193 mg/dL
Lupus Anticoagulation Ratio	1.10	< 1.21
Beta-2 Glycoprotein IgG	7.3 U/ml	0.0 – 20.0 U/ml
Beta-2 Glycoprotein IgM	1.3 U/ml	0.0 – 20.0 U/ml
Anticardiolipin IgG	< 9.4 GLP	0.0 – 14.9 GLP
Anticardiolipin IgM	< 9.4 GLP	0.0 – 14.9 GLP
Chemokine ligand 9 (CXCL9 )	1,947 pg/mL	<647 pg/mL
Soluble IL-2 Receptor	1,790.7 pg/mL	175.3 – 858.2 pg/mL
Urine analysis	Unremarkable	NA

A chest X-ray (CXR) demonstrated bibasilar airspace opacities with small bilateral pleural effusions.

**Figure 2:**
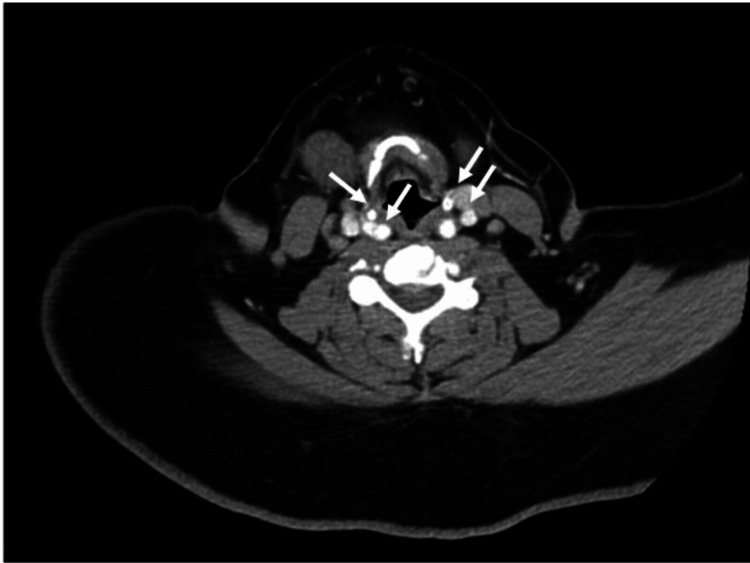
CT of the soft tissue head and neck showing scattered enlarged cervical lymph nodes without bulky lymphadenopathy.

A CT scan of the head and neck showed evidence of scattered enlarged cervical lymph nodes without bulky lymphadenopathy (Figure 2).

**Figure 3:**
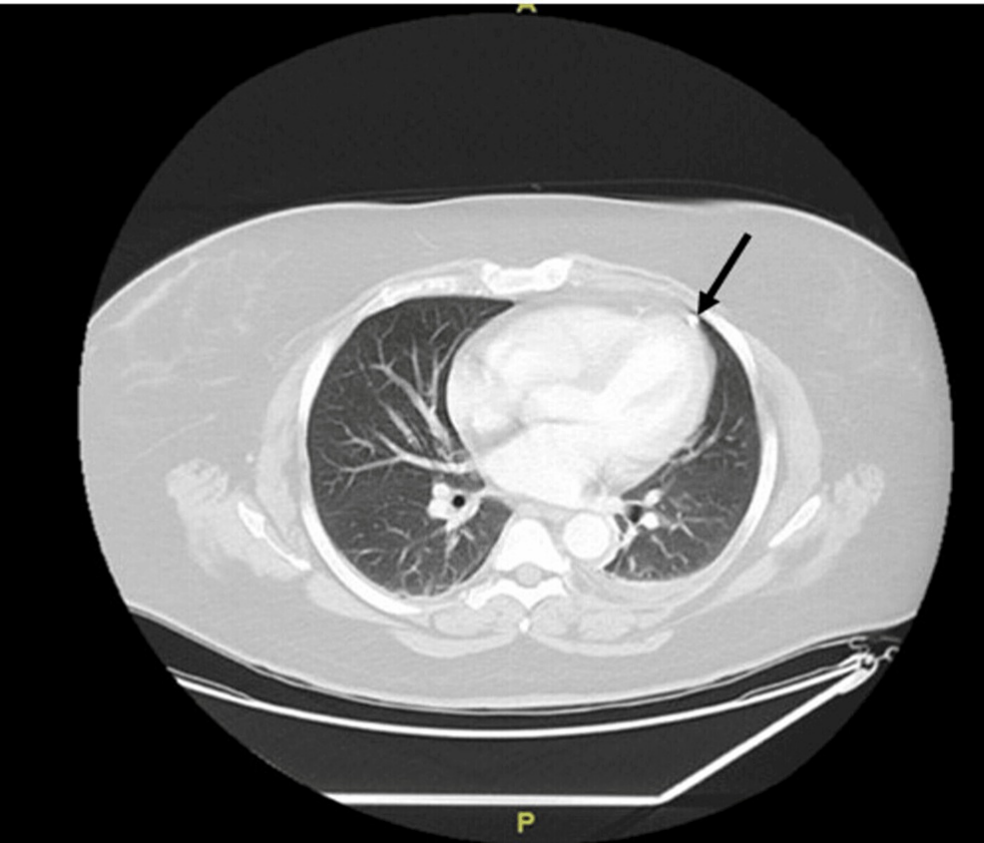
CT of chest with intravenous contrast showing mild dependent airspace disease with evidence of a calcified granuloma in left upper lung.

CT of the chest showed mild dependent airspace disease with evidence of a calcified granuloma in the left upper lung (Figure 3). The spleen was normal in size on the CT scan of the abdomen.

As the fever episodes persisted, an extensive infectious disease work-up was performed, revealing only two indeterminate Quantiferon TB tests. The patient was evaluated by infectious disease who recommended against treatment for latent tuberculosis infection due to low clinical suspicion. A connective tissue disease work-up was negative (Table 1). Despite several days of intravenous ketorolac, the febrile episodes persisted. Repeat blood investigations on day 6 of hospitalization demonstrated a ferritin level of 12,189 ng/mL, CRP 198.63 mg/L, and ESR 82 mm/H. A diagnosis of adult-onset Still's disease (AOSD) was made based on the Yamaguchi criteria which entails four major criteria, including fever >39 C lasting 7 days or longer, arthralgia or arthritis lasting 14 days or longer, typical rash, and WBC count>10,000 with >80% neutrophils. The minor criteria include: Sore throat, hepatosplenomegaly, lymphadenopathy, abnormal liver function tests, and negative rheumatoid factor and anti-nuclear antibody. The exclusion criteria include infections, malignancies, and other rheumatic diseases. Prednisone 60 mg daily was initiated. Febrile episodes ceased to occur following prednisone initiation and inflammatory markers began to downtrend. Canakinumab was then initiated shortly after hospital discharge, and prednisone was gradually tapered until eventual discontinuation. Canakinumab was continued for 6 months. No further episodes have occurred since canakinumab initiation. Follow-up blood work is shown in Table 2.

**Table 2:** Blood investigation two weeks after starting canakinumab. CRP: C-Reactive Protein; ESR: Erythrocyte Sedimentation Rate

	Results	Normal Range
WBC	16.6 x 10^9^/L	3.5 – 11.0 x 10^9^/L
Absolute Segs	13.9 x 10^9^/L	1.5 – 7.5 x 10^9^/L
Ferritin	798 NG/mL	10 – 120 NG/mL
CRP	18.41 mg/L	0.00 – 10.00 mg/L
ESR	57 mm/hr	0 – 30 mm/hr

## Discussion

Adult-onset Still's disease (AOSD) is an autoinflammatory condition characterized by the inappropriate activation of the innate immune system. The main mediators involved in the pathogenesis of AOSD include interleukin (IL)-1, IL-6, and IL-18 [9]. Blocking the receptors of these interleukins has shown efficacy in treating AOSD [10]. On the other hand, SARS-CoV-2 infection has been reported to activate the innate immune system, leading to an increase in neutrophils, mononuclear phagocytes, and natural killer cells, and a decrease in T cells, including CD4+ and CD8+ cells [11]. The inflammatory response to SARS-CoV-2 involves a massive release of interleukins, including IL-1 and IL-6 [11].

The activation of the innate immune system can manifest as macrophage activation syndrome (MAS) clinically. In our case, we observed positive results for chemokine ligand 9 (CXCL9) and soluble IL-2 receptor, which are among the features included in the Delphi International Survey for diagnosing macrophage activation syndrome (MAS) [12]. However, it is important to note that our patient did not exhibit the main features proposed for a diagnosis of MAS, such as abnormalities in the complete blood count, hypertriglyceridemia, hypofibrinogenemia, central nervous system dysfunction, or increased liver enzymes [12]. CXCL9 can be also positive in patients with graft-versus-host disease. Considering the presence of fever and systemic symptoms coupled with marked hyperferritinemia, it is likely that our patient exhibited the systemic phenotype of AOSD. However, there is currently a lack of clear data to predict or compare the phenotypes of AOSD following COVID-19 infection.

Given that both COVID-19 and AOSD involve a massive release of cytokines, it is hypothesized that SARS-CoV-2 infection might trigger AOSD. However, limited data supports this theory. Only four case reports suggest the induction of AOSD by SARS-CoV-2 infection (Table 3). The data from these cases is inconsistent, as AOSD symptoms started more than 2 weeks after COVID-19 positivity in two cases, compared to less than one week in the other two cases. Corticosteroids were effective in two cases, while the other two cases experienced a relapse after discontinuation of corticosteroids. Canakinumab was utilized in our case to treat the systematic involvement of the disease as well as to proactively prevent the development of chronic arthritis, capitalizing on the concept of the "window of opportunity" [10]. Based on data from systemic juvenile arthritis, IL-1 plays a pivotal role in innate and adaptive immunity of acute febrile systemic juvenile arthritis via promoting inflammation in an antigen-dependent manner through activation of leukocytes, endothelium, and resident tissue lineages [10]. IL-1 activation can lead to autoimmune T-cell-driven arthritis by inhibiting the efficacy of Treg cells and directly promoting Th17 differentiation (10). Therefore, early IL-1 blockade, compared to later IL-1 inhibitor initiation, is associated with improved outcomes [13, 14].

**Table 3:** Literature cases describing the occurrence of adult-onset Still's disease following COVID-19 infection.

Case	Onset of symptoms	Diagnosis	Treatment	Follow-up
Alshablan et al. [18]	8 weeks after resolution of COVID-19 symptoms	4 major 4 minor	Intravenous methylprednisolone then oral prednisolone	Not Reported
Bamidis et al. [19]	15 days after negative COVID-19 test	4 major 3 minor	Failure of high dose prednisolone Anakinra subcutaneously 100 mg/day	Symptoms subsided and laboratory values normalized during the following months after treatment
Ibanez et al. [20]	3 days after positive COVID-19 test	3 major 5 minor	Methylprednisolone 60 mg/day	Relapse after tapering corticosteroids
Alzuhaily et al. [21]	1 week after positive COVID-19 test	4 major 3 minor	High-dose oral prednisolone	Symptom free after 1 year
Our case	One month after positive COVID-19 test	3 major 2 minor	High-dose oral prednisolone Canakinumab	Symptom free after 2 weeks

Furthermore, AOSD has also been reported to occur following COVID-19 vaccination [15]. The incidence of AOSD has been reported to be 4.82 times higher in the COVID-19 vaccinated cohort [15]. Various types of vaccines, including BNT162b2 mRNA and ChAdOx1 nCoV-19, have been associated with AOSD (15). Notably, AOSD flares have been observed after the second dose of the COVID-19 vaccine, likely due to a strong immune response [16].

Nirmatrelvir/ritonavir has shown favorable outcomes and an acceptable safety profile among patients with systemic autoimmune rheumatic diseases [6]. While a rebound phenomenon of COVID-19 has been described as a side effect of nirmatrelvir/ritonavir [17], there have been no reports of induction of autoimmune diseases, including AOSD.

## Conclusions

In conclusion, COVID-19 infection or medications used to treat COVID-19 may alter the immune system, leading to a massive release of cytokines, many of which are involved in the pathophysiology of AOSD. Although rare, AOSD can be diagnosed after a COVID-19 infection. The exact mechanism is unknown but is thought to involve alteration of the innate immune system. Glucocorticoids are often used in AOSD treatment but the disease may sometimes flare upon tapering glucocorticoids. Therefore, steroid-sparing agents such as IL-1 inhibitors, have been used with positive outcomes.
